# Compensatory intestinal antibody response against pro-inflammatory microbiota after bariatric surgery

**DOI:** 10.1080/19490976.2022.2031696

**Published:** 2022-02-07

**Authors:** Torsten P.M. Scheithauer, Mark Davids, Maaike Winkelmeijer, Xanthe Verdoes, Ömrüm Aydin, Maurits de Brauw, Arnold van de Laar, Abraham S. Meijnikman, Victor E.A. Gerdes, Daniël van Raalte, Hilde Herrema, Max Nieuwdorp

**Affiliations:** a Department of (Experimental) Vascular Medicine, Amsterdam University Medical Center (UMC), Amsterdam, The Netherlands; b Department of Surgery, Spaarne Gasthuis, Hoofddorp, The Netherlands; c Diabetes Center; Department of Internal Medicine, Amsterdam University Medical Center (UMC), Amsterdam, The Netherlands

**Keywords:** Immunoglobulin, gut microbiome, lipopolysaccharide, flagellin, bariatric surgery

## Abstract

Obesity and type 2 diabetes (T2D) are growing burdens for individuals and the health-care system. Bariatric surgery is an efficient, but drastic treatment to reduce body weight, normalize glucose values, and reduce low-grade inflammation. The gut microbiome, which is in part controlled by intestinal antibodies, such as IgA, is involved in the development of both conditions. Knowledge of the effect of bariatric surgery on systemic and intestinal antibody response is limited. Here, we determined the fecal antibody and gut microbiome response in 40 T2D and non-diabetic (ND) obese individuals that underwent bariatric surgery (N = 40). Body weight, fasting glucose concentrations and inflammatory parameters decreased after bariatric surgery, whereas pro-inflammatory bacterial species such as lipopolysaccharide (LPS), and flagellin increased in the feces. Simultaneously, concentrations of LPS- and flagellin-specific intestinal IgA levels increased with the majority of pro-inflammatory bacteria coated with IgA after surgery. Finally, serum antibodies decreased in both groups, along with a lower inflammatory tone. We conclude that intestinal rearrangement by bariatric surgery leads to expansion of typical pro-inflammatory bacteria, which may be compensated by an improved antibody response. Although further evidence and mechanistic insights are needed, we postulate that this apparent compensatory antibody response might help to reduce systemic inflammation by neutralizing intestinal immunogenic components and thereby enhance intestinal barrier function after bariatric surgery.

## Introduction

Obesity^1^ and type 2 diabetes (T2D)^2^ are pandemics that challenge health-care systems worldwide. The World Health Organization (WHO) defines obesity as excessive fat accumulation that can impair the individual’s health and may lead to comorbidities, such as glucose intolerance.^1^ Treatment strategies aim to reduce body weight via lifestyle changes that include a balanced diet and an increase in physical activity.^1^ However, if individuals are not able to reduce their body weight, bariatric surgery may be an efficient, but drastic intervention to overcome severe obesity and its long-term consequences.^3^

Roux-en-Y gastric bypass (RYGB) reduces the volume of the stomach while bypassing the proximal part of the intestine and changing the route of the ingested food.^4^ Long-term studies report a substantial reduction in body weight and indicate that RYGB is an efficient way to treat T2D^3^. Short- and long-term studies report a major improvement in glucose metabolism after RYBG.^3^ However, bariatric surgery includes a drastic rearrangement of the intestinal route that comes with shifts in the gut microbiome composition.^5^

The gut microbiome is a collection of microbial organisms, including bacteria, fungi, protozoa, and viruses in the gastrointestinal tract.^6^ A plethora of studies have highlighted the involvement of the gut microbiome in numerous diseases like obesity and T2D^6^. An increase in pro-inflammatory bacterial species, including proteobacteria, and bacterial components, such as lipopolysaccharide (LPS) and flagellin may be partially responsible for the so-called low-grade inflammation observed in metabolic diseases.^6–8^ A pro-inflammatory gut microbiome may also trigger an immune response via bacterial translocation or influx of LPS and flagellin into the blood circulation toward metabolically active tissues.^6,7^ Nevertheless, convincing evidence for this concept in humans is still lacking.

In line, an intact intestinal epithelium, a functional immune system, and a symbiotic gut community are the major defense systems to prevent leakage of intestinal bacterial strains and/or bacterial components into the blood circulation.^6^ In obese mouse models, the intestinal epithelium is more permeable, and the intestinal immune response is insufficient to capture pro-inflammatory bacterial molecules.^7,8^ In particular, intestinal B cells produce less immunoglobulin A (IgA) in an obese setting, which is a sign of an inadequate immune response toward a pro-inflammatory gut microbiome.^8^

In the current paper, we report on the intestinal and systemic immune response after bariatric surgery in paired samples of 40 severely obese non-diabetic (ND) and T2D individuals from our BARIA cohort.^9^ RYGB surgery induced a shift toward a pro-inflammatory gut microbiome. Interestingly, systemic low-grade inflammation was reduced after surgery; albeit to a different extent in ND and T2D patients.^9^ Intestinal IgA levels against pro-inflammatory bacteria and bacterial components increased after bariatric surgery. We postulate that increased intestinal IgA levels upon bariatric surgery neutralize immunogenic bacteria and bacterial components, which aids in lowering the inflammatory tone. However, some pro-inflammatory bacteria might evade the IgA response after surgery.

## Results

To investigate the antibody and gut microbiome response after bariatric surgery, we included a total of 40 obese individuals with or without T2D from our BARIA cohort.^9^ We matched individuals according to age, sex, and body mass index (Table S1) and measured antibody concentrations and gut microbiome composition before as well as one year after surgery. As expected, body weight and glucose response improved after surgery in both groups (Table 1).

**Table 1.** Comparison of characteristics before and after surgery

	All (N = 40)			ND (n = 20)			T2D (n = 20)		
	Pre	Post	p-value	Pre	Post	p-value	Pre	Post	p-value
BMI (kg/m^2^)	40.7 (4.8)	29.6 (11.3)	<0.0001	40.8 (5.4)	29.4 (10.9)	<0.0001	40.1 (4.3)	29.8 (11.9)	<0.0001
HbA1c (%)	6.50 (1.3)	5.51 (2.3)	<0.0001	5.64 (0.4)	5.21 (2.0)	0.0003	7.39 (1.2)	5.83 (2.6)	<0.0001
CRP (mg/mL)	6.56 (7.1)	3.82 (11.7)	<0.0001	5.25 (5.1)	1.48 (1.6)	<0.0001	7.92 (8.6)	6.54 (16.6)	0.0019
Leukocytes (10^9^/L)	7.45 (2.2)	6.38 (3.1)	<0.0001	6.58 (1.5)	5.7 (2.5)	0.0136	8.35 (2.4)	7.18 (3.7)	0.0024
Fasting glucose (mmol/L)	7.51 (2.7)	5.80 (2.29)	<0.0001	5.75 (0.5)	4.98 (1.1)	<0.0001	9.33 (2.78)	6.77 (3.05)	0.0002
LBP (ug/mL)	23.5 (10.7)	15.5 (6.6)	<0.0001	22.4 (11.3)	14.1 (5.0)	0.0004	24.7 (9.9)	16.8 (7.6)	0.0134
Serum lipocalin-2 (ng/mL)	33.5 (11.5)	25.4 (8.6)	<0.0001	35.4 (12.1)	27.6 (8.7)	0.0007	31.8 (10.9)	23.4 (8.3)	<0.0001

Obese individuals with or without Type 2 diabetes underwent Roux-en-Y gastric bypass. The mean (standard deviation) results are shown. Values before and after surgery were compared. Statistical analysis was performed using paired t-test or Wilcoxon signed-rank test. Abbreviations: ND, no diabetes; T2D, Type 2 diabetes; BMI, body mass index; HbA1c, glycated hemoglobin; CRP, c-reactive peptide; LPS, lipopolysaccharide; OD, optical density; LBP, LPS binding protein.

### Bariatric surgery improves systemic inflammation

**Figure 1.**
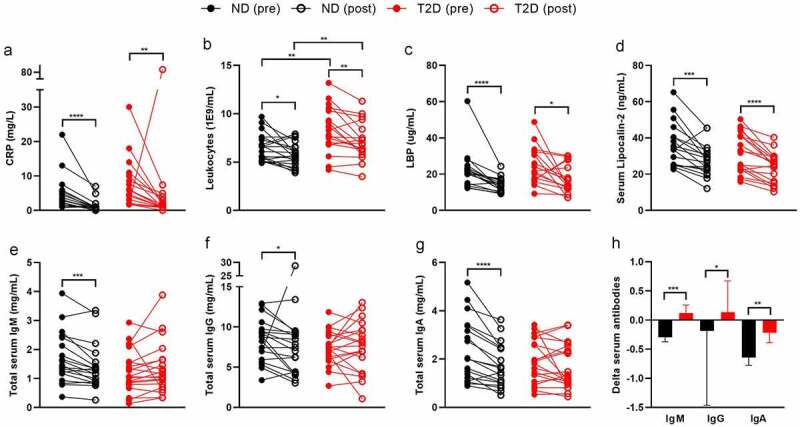
Bariatric surgery improves systemic inflammation in humans.

Systemic inflammation decreased after surgery in both groups (Table 1). For example, CRP (Figure 1a) and blood leukocytes (Figure 1b) decreased in all individuals. Translocation of bacterial components is associated with low-grade inflammation in obese humans.^7,10^ As a proxy for translocation of bacterial components, we measured bacterial LPS, flagellin, and LPS binding protein (LBP) in the blood circulation. Although serum LPS and flagellin did not significantly differ between the time points (Table S2), LBP decreased upon surgery in both groups (Figure 1c). Similarly, serum lipocalin-2, a player in inflammation and gut microbiota homeostasis^11^ decreased after surgery (Figure 1d).

Participants of the BARIA cohort underwent bariatric surgery. Systemic inflammation was measured in ND and T2D before as well as one year after surgery (N = 40). CRP (A), Leukocyte count (b), LBP (c), serum lipocalin-2 (d), and total antibodies (e, f, g) decreased in ND and partly in T2D. Delta serum antibodies changed differently after surgery between ND and T2D (h). Individual values (a-g) or mean with SEM (h) are shown. Statistical analysis was performed using Wilcoxon (a-g) and Mann-Whitney (b, h) tests: *p < .05, **p < .01, ***p < .001, ****p < .0001. Abbreviations: ND, no diabetes (ND); T2D, Type 2 diabetes; LBP, lipopolysaccharide-binding protein; OD, optical density; Ig, immunoglobulin; CRP, c-reactive peptide.

Low-grade inflammation and endotoxemia are associated with T2D.^7,10^ However, many studies compare lean controls to obese T2D individuals, where obesity is a major confounding factor.^12^ Here, we matched severely obese ND to severely obese T2D individuals. Age, sex, and BMI did not differ at both time points (Table S1). As expected, fasting glucose and glucose response during a mixed meal test (Figure S1) and HbA1c, a marker for long-term glucose control, were increased in T2D (Table S1). Further, we noticed a numerical increase in C-reactive peptide (CRP, Figure 1a) and a significantly higher leukocyte count in T2D (Figure 1b), suggesting a low-grade inflammation in this group (Table S1). These differences persisted after bariatric surgery.

Translocation of bacterial components has been associated with T2D,^13^ yet, serum LPS, flagellin, and LBP did not differ between the two matched groups (Table S1). However, we observed a significant positive correlation between fasting glucose and serum LPS as well as serum flagellin (Figure S2), indicating a relation between disturbed glucose tolerance and translocation of bacterial components. This correlation did not persist after surgery, potentially due to the reduction of all glucose parameters, endotoxemia, and BMI. A reduction in body weight, particularly adipose tissue, has been associated with reduced inflammation and endotoxemia.^14,15^ Most of the participants had a BMI below 30 after surgery and therefore were categorized as overweight rather than obese.

Intestinal and circulating antibodies serve an important role in preventing translocation of bacterial components or neutralize immunogenic effects.^16^ T2D individuals had slightly lower serum immunoglobulin (Ig)G, IgA, and IgM levels compared to ND at both time points (Table S1). Serum IgA, IgM, and IgG decreased in ND after surgery, highlighting that the inflammatory tone improved (Table S2, Figure 1e-g). In individuals with T2D, serum IgM numerically increased and IgG levels did not change after surgery. Treatment-induced changes in serum antibody levels were significantly different in ND compared to T2D (Figure 1h), suggesting that bariatric surgery is less effective in reducing inflammation in diabetic individuals. Serum antibodies against intestinal-derived compounds, such as LPS and flagellin did not change significantly after RYGB surgery in both groups except for an increase in IgA against LPS (Table S2).

We next questioned whether antibody production from B cells was different between phenotypes and changed after surgery. Therefore, we isolated peripheral B cells from a subset of 12 individuals (at baseline and post-surgery) and measured B cell antibody production *ex vivo*. To do so, memory B cells with either of the immunoglobulin subsets (IgG, IgM, and IgA) were sorted and differentiated into antibody-produced plasma cells using IL-21 and CD40L. In line with serum antibody levels, B cells derived from individuals with T2D produced less antibodies at both time points than B cells derived from ND individuals (Table S3). The B cell subsets were not significantly different between both groups but changed after surgery. For example, there were less naïve B cells, potentially due to preferred differentiation into other effector B cells. Since we only had B cell subsets relative to CD19+ cells and not total blood B cells, we are unable to provide certainty on this suggestion. Furthermore, B cells derived from post-bariatric surgery individuals produced more antibodies *ex vivo* (Table S4).

### RYGB Surgery induces bloom of typical pro-inflammatory bacteria

**Figure 2.**
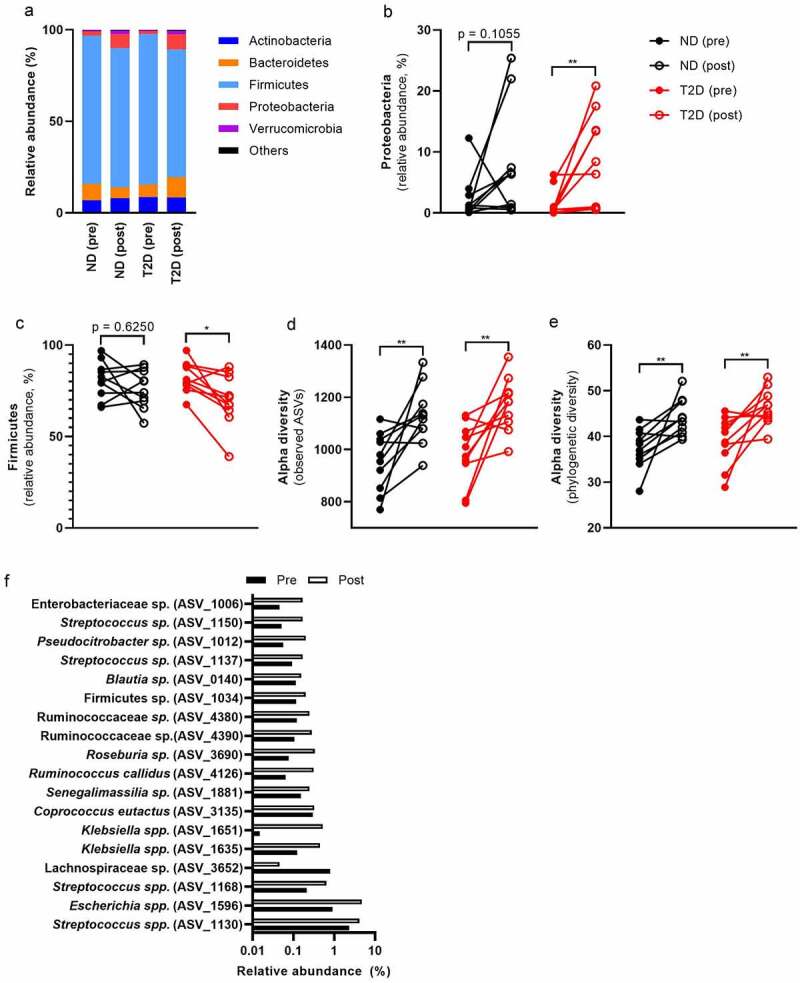
Bariatric surgery induces a more pro-inflammatory gut microbiome one year after RYGB surgery.

Despite the decrease in systemic inflammation after bariatric surgery, we observed an increase from 1.93% to 8.04% in pro-inflammatory Proteobacteria (Figure 2a–b, Table S5). This increase was mostly at the expense of Firmicutes (Figure S3A and S3B), which decreased after surgery in relative abundance (Figure 2c, Table S5). We did not observe major differences in microbial composition between ND and T2D (Table S6), potentially due to our small sample size and extensive group matching. Nevertheless, individuals with T2D had a lower abundance of *Streptococcus salivarius* than ND (Table S6). Interestingly, *S. salivarius* has been discussed as an anti-inflammatory (orally derived) bacterial strain.^17,18^ A decrease in that bacterium might contribute to the higher inflammatory tone of T2D.

Participants of the BARIA cohort underwent bariatric surgery. Intestinal gut microbiota was measured in homogenized gut bacteria of ND and T2D humans. Samples were taken before and one year after surgery. The gut microbiota changes at the phylum level after surgery (a). Proteobacteria (b) increased, whereas Firmicutes (c) decreased after surgery in relative abundance. Alpha diversity increased after surgery in both groups (d, e). In total, 18 ASVs with at least 0.1% abundance changed significantly after surgery (f). The mean (a, f) and every individual separately (b-f) is shown. Statistical analysis was performed using Wilcoxon test (b-f): *p < .05, **p < .01. Abbreviations: ND, no diabetes; T2D, Type 2 diabetes; ASV, amplicon sequence variant.

We found that alpha diversity increased after surgery in both groups (Figure 2d-e). Pre- and post-surgery microbiomes could clearly be separated in both groups (PERMANOVA, p = .005, R2 = 0.041, Figure S4). In this regard, 42 significant amplicon sequence variants (ASVs) changed after surgery (Table S9). Of those, 18 ASVs had an abundance of more than 0.1% (figure 2f) with ASV_1596 (*E. coli*, 2.8%) and ASV_1130 (*S. salivarius*, 3.2%) as the most abundant bacteria that changed after surgery. Mostly facultative anaerobic bacteria increased that are able to adapt to the changing environment after RYGB surgery. For example, eight of these ASVs belong to the family Enterobacteriaceae that, as a group, increased in ND and T2D (Table S5). Similarly, 11 ASVs belong to the facultative anaerobe genus *Streptococcus* that increased after surgery in both groups (Table S5). An increase in oxygen^19^ or more easily fermented dietary components reach the colon due to the changed route of the intestine after RYGB surgery. That might result in altered relative abundance of facultative anaerobic bacteria.

**Figure 3.**
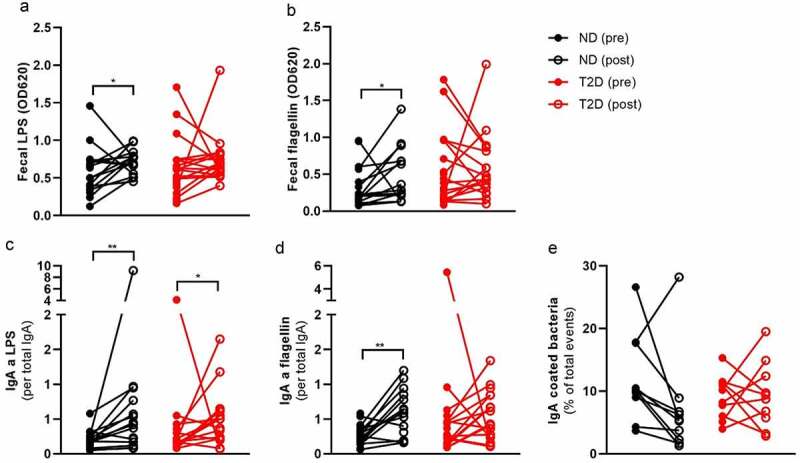
Bariatric surgery increases fecal LPS, flagellin and IgA against both bacterial compounds.

Despite the improvement in systemic inflammation (CRP, blood leukocytes, serum IgA), we observed an increase in pro-inflammatory fecal LPS and flagellin abundance after RYGB surgery (Figure 3a–b, Table S7), without obvious differences between ND and T2D (Table S8). Increased LPS after RYGB surgery is likely derived from the increase in LPS-carrying Proteobacteria. The intestinal IgA response is important to neutralize bacterial ligands in the intestine.^16^

Participants of the BARIA cohort underwent bariatric surgery. Intestinal antibody was measured in ND and T2D before as well as one year after surgery. Intestinal LPS (a), flagellin (b), IgA against LPS (c) and IgA against flagellin (d) increased after surgery. IgA coated bacteria did not change significantly (e). Wilcoxon test was used: *p < .05, **p < .01. Abbreviations: ND, no diabetes; T2D, Type 2 diabetes; LBP, lipopolysaccharide-binding protein; OD, optical density; Ig, immunoglobulin.

### Typical pro-inflammatory bacteria evade IgA coating after RYGB Surgery

Previously, it was reported that RYGB surgery increased total fecal IgA one month after surgery.^20^ However, there are no reports of IgA coated bacteria or compound-specific fecal IgA. We did not find any significant changes in total fecal IgA one year after surgery (Table S7). Similarly, we did not observe changes in the fecal lipocalin-2 (Table S7), which is a marker for intestinal inflammation.^21^ However, we observed an increase in LPS and flagellin-specific IgA (Figure 3c–d), which is potentially a response toward the more pro-inflammatory gut microbiome. We postulate that this might also lead to a higher IgA coating of the gut microbiome. Therefore, we labeled fecal bacteria with an antibody against IgA and sorted the IgA-coated as well as IgA-uncoated fraction. Surprisingly, we found a non-significant decrease in IgA coating after surgery, suggesting that other parts of the intestinal barrier function buffer the increase in pro-inflammatory bacteria (Table S7, Figure 3e). There was no change in total fecal IgM. Fecal IgM against LPS and flagellin were barely detectable.

**Figure 4.**
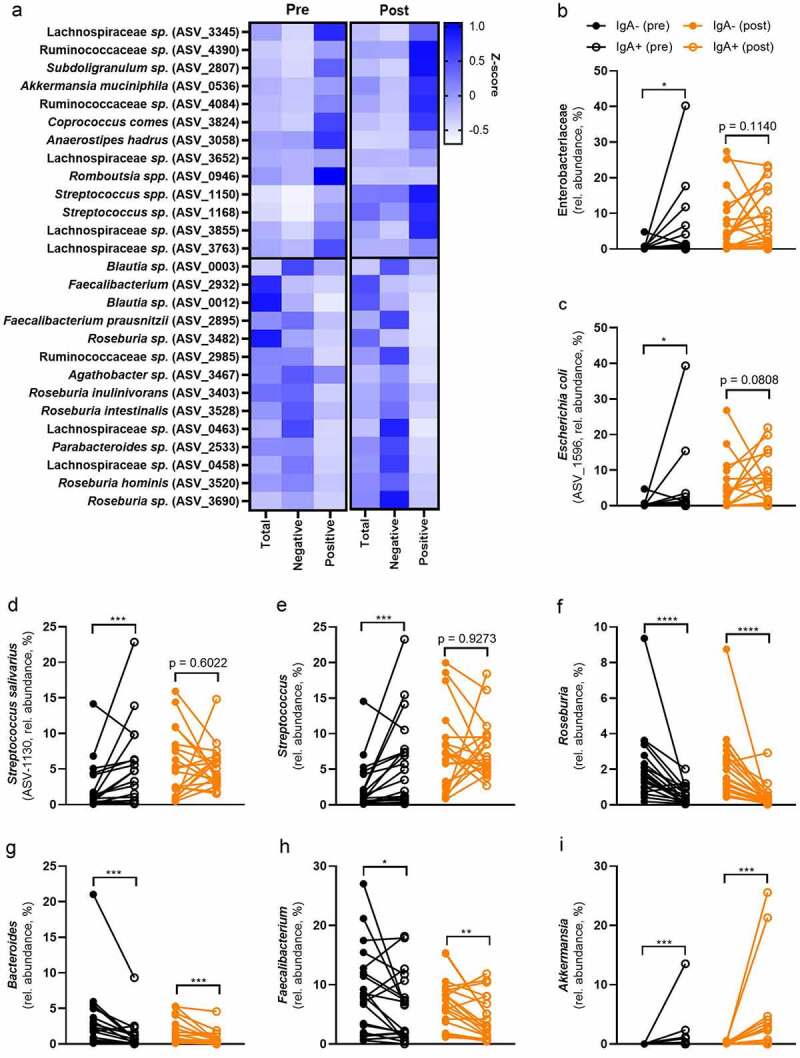
Bariatric surgery changes the IgA coating of intestinal bacteria.

Enterobacteriaceae^22^ and *Streptococcus^23^* include several pro-inflammatory bacteria that need to be contained in the intestine to prevent infections. We sorted IgA coated bacteria and sequenced their 16s rRNA. We did not observe any significant differences between IgA-coated bacteria in ND compared to T2D fecal samples (Figure S5). Therefore, we combined both groups and used linear mixed effect models to discern taxa that were enriched in either IgA negative or positive fractions and potential effects of surgery on IgA coating (Table S9). We then observed a clear separation between IgA positive and negative fractions at both time points (Figure S5). We found 77 ASVs that were significantly enriched in either of the IgA fractions and had diverse mean relative abundance (0.003% to 3.84%, Table S9). To simplify the results, we are going to elaborate on the relative abundance and IgA coatings of the most abundant ASVs as well as bacterial groups (mean relative abundance >0.1%, Figure 4a).

Participants of the BARIA cohort underwent bariatric surgery. IgA coating of fecal bacteria was measured in ND and T2D humans before as well as one year after surgery. In total 27 ASVs that have a relative abundance of at least 0.1%, were differentially coated with IgA (A). Bacterial groups and specific ASVs are either enriched in the IgA positive or negative fraction (B-I). Statistical analysis was performed using Wilcoxon test (B-I): *p < .05, ***p < .001, ****p < .0001. Abbreviations: ND, no diabetes; T2D, Type 2 diabetes; ASV, amplicon sequence variant.

The phylum Proteobacteria was numerically enriched in the IgA-coated fraction (Figure S3C), which suggests that the majority of ASVs belonging to this phylum are IgA-coated. Of the eight significant ASVs in Proteobacteria, all were enriched in the IgA-coated fraction (p < .05). Similarly, the family Enterobacteriaceae was enriched in the IgA-positive fraction before surgery, which was surprisingly not significant after surgery (Figure 4b). Therefore, we can confirm results from previous studies that typical pro-inflammatory bacteria, belonging to the group of Proteobacteria as well as Enterobacteriaceae, are coated with IgA.^16,24^

As reported before, the IgA coating seems to be at least genus, species, or strain-specific.^24^ Higher taxonomic ranks showed a diverse enrichment in either of the IgA fractions (Table S9). For example, out of the 41 ASVs belonging to the family Lachnospiraceae, 14 were enriched in the IgA coated fraction and 27 in the IgA negative fraction. In contrast, genera, such as *Roseburia* and *Streptococcus,* were significantly enriched in either of the fractions. This suggests that there are shared bacterial wall patterns or intestinal niches (*e.g*., close to the epithelial wall or in the mucus layer) that determine if a bacterium gets IgA coated.

ASV-1596 that includes *Escherichia coli*, was significantly enriched in the IgA-coated fraction before surgery, but not significant after surgery (Figure 4c). Similarly to the whole group of Proteobacteria, *E. coli* relative abundance increased after surgery (0.92% to 4.80%, Table S5) and had the highest abundance within that phylum. There was no difference between ND and T2D (Table S6). To simplify the vast number of significant findings, we will focus only on the most abundant ASVs in the total bacterial fraction (> 0.1%). The majority of significant ASVs that belong to the phylum Proteobacteria falls below that cutoff.

The genus *Streptococcus* includes several facultative anaerobes and opportunistic pathogens.^23^ All five significant ASVs were enriched in the IgA-positive fraction (Table S9). ASV-1130, which includes *S. salivarius*, was the most abundant species of the genus in the present study. The relative abundance significantly increased from 2.35% to 4.18% after surgery (Table S5). Before surgery, *S. salivarius* was highly enriched in the IgA positive fraction (Figure 4d). After surgery, it was not significantly different, which might indicate either a more tolerant immune response toward bacteria with beneficial activities or the evasion of the immune response after surgery. Surprisingly this accounts for the whole genus *Streptococcus*, which was highly enriched in the IgA-positive fraction before surgery, but not differently abundant in either of the fractions after surgery (Figure 4e).

*Roseburia* is a highly abundant genus that includes several beneficial bacteria.^25^ The mean relative abundance slightly decreased after surgery from 2.56% to 2.17% (Table S5) without any differences between ND and T2D (Table S6). *Roseburia* is strongly enriched in the IgA negative fraction at both time points (figure 4f). All the 13 significant ASVs belonging to this genus were enriched in the IgA negative fraction. It included *R. intestinalis* (ASV-3528), which was highly abundant in our data set (7.81%) and has several beneficial effects on the host.^26^

Similarly, *Bacteroides* includes several beneficial bacteria that are able to produce *e.g*., short-chain fatty acids (SCFAs). There was a non-significant reduction after surgery and no difference between the two groups (Table S5 and S6). The whole group of *Bacteroides* was enriched in the IgA negative fraction at both time points (Figure 4g). This suggests that the host displays a tolerance against beneficial bacteria.

Recently, we reported that ASVs of *Faecalibacterium* were enriched in IgA-positive as well as the IgA-negative fraction before antibiotic treatment.^24^ Interestingly, in the present study all six significant ASVs of *Fecalibacterium* were in the IgA-negative fraction. Indeed, the whole genus was enriched in the IgA negative fraction (Figure 4h), supporting the notion that beneficial bacteria do not elicit an inflammatory response but an immune tolerance. The relative abundance did not change after surgery (Table S5), nor was there a difference between ND and T2D (Table S6).

*Akkermansia muciniphila* has several important functions for the host,^27^ but may also induce an inflammatory response.^28^ In line with our previous findings,^24^ *A. muciniphila* was IgA-coated in the present study. The genus *Akkermansia*, which was mostly represented by ASV-0536 (*A. muciniphila*), was highly enriched in the IgA positive fraction (Figure 4i). This genus increased after surgery (Table S5) and did not show a difference between ND and T2D.

## Discussion

In the present study, we addressed the intestinal and systemic antibody response in relation to the gut microbiome after bariatric surgery. We included severely obese individuals with or withoutT2D from the BARIA cohort.^9^ We show that individuals with T2D have a higher inflammatory tone than non-diabetic controls, which improved after surgery. Furthermore, we found an increase in intestinal IgA and IgM against LPS as well as flagellin, which we hypothesize to be a response to the pro-inflammatory gut microbiome after surgery. Proteobacteria were particularly increased at this time point, whereas Firmicutes were decreased. Most of the typical pro-inflammatory bacteria were coated with IgA at both time points. Surprisingly, some of these bacteria seem to evade this IgA response after surgery.

It is well established that T2D individuals have a higher chronic low grade inflammatory tone than healthy controls.^6^ Indeed, the T2D individuals in the present study had higher blood leukocytes and a numerical increase in CRP. Both decreased after surgery, but remained higher in T2D compared to ND individuals. Several factors seem to be involved in the inflammatory response in T2D. First, higher glucose levels, and other excessive nutritional factors in the blood circulation can induce an inflammatory cascade.^29^ Glucose values improved in both groups after RYGB surgery, but were still elevated in T2D. That might explain the higher inflammatory tone before and after surgery. Second, gut-derived LPS can induce inflammation in T2D^13^. We did not find increased LPS levels in T2D, which might be due to the extensive matching of the present groups. LBP, as a proxy for LPS exposure, was increased numerically when comparing ND to T2D. In addition, we found a positive correlation between blood glucose concentration and serum LPS as well as flagellin. This suggests that there is a relationship between glucose values and endotoxemia. Indeed, Thaiss *et al*. (2018) proposed a mechanism how hyperglycemia reduces the intestinal barrier function.^30^ Therefore, there is a strong association between inflammation, gut microbiome and hyperglycemia.^6^

Many studies focus on macrophages and T cells in relation to inflammation in T2D^29^, whereas the B-cell response is rather neglected. We found a numerical decrease in the B-cell response between T2D and ND. We can only speculate that reduced systemic antibody levels, which are still in a healthy range, may hint toward a less efficient immune response against pro-inflammatory bacterial components such as LPS. More research and more sensitive methods are necessary to further decipher the antibody response in T2D, particularly against gut-derived compounds.

Interestingly, the antibody response in T2D individuals after RYGB surgery differed from the response in ND. Whereas systemic IgA decreased in both groups, IgM and IgG slightly increased in T2D. If the hypothesis is true that T2D individuals have reduced antibody levels, then bariatric surgery might help to improve the antibody response to a healthier status. Indeed, there was a higher antibody production in B cell cultures *ex vivo*. Of note, these cultures were treated with cytokines to stimulate antibodies. In particular, IL-21, which we use in our B cell culture, dose dependently increases antibodies *in vitro*.^31^ Therefore, we cannot compare *ex vivo* culture with total plasma antibody levels. Nevertheless, we believe that this increase further highlights a healthier immune response after surgery. However, we cannot exclude that technical artifacts may be responsible for this effect since B cells were cultured freshly and there is one year between both time points.

Next, we found that fecal LPS and flagellin increased after RYGB surgery. This increase may be attributed to an increase in Proteobacteria in the fecal microbiome as RYGB surgery induces a drastic change in the intestinal anatomy. Studies suggest that the shortened path from the stomach toward the large intestine, where a major portion of the gut microbiome resides, may introduce more oxygen.^19^ Other factors include different delivery routes and concentrations of various bile acids,^32^ different diet patterns,^32^ proton pump inhibitor use after surgery, as well as a higher pH in the intestine.^33^ One adaptation to these observed changes is a higher antibody production against the pro-inflammatory microbiome after RYGB surgery.

A previous study reported an increase in total fecal IgA one month after surgery.^20^ In contrast, we did not find an increase in total fecal IgA, which might be due to the fact that we measured antibodies one year after surgery. Furthermore, fecal IgA is highly variable and standardized methods as well as concentrations are not clearly defined at this point in time. More research is needed to better map ‘healthy’ intestinal IgA levels and to create reference ranges as exist for systemic antibodies. Nevertheless, we found higher antibody levels against LPS and flagellin after surgery, which suggests that the intestinal barrier function is improving. Further, there was an increase in systemic IgA against LPS, similar to the intestinal IgA against LPS. Thereby, increased levels of fecal LPS and flagellin can potentially be neutralized, which aids in maintaining intestinal barrier function after RYGB surgery.^34–36^

IgA coating of intestinal bacteria is an important tool of the host to prevent bacteria from interacting with the epithelium or even pass the epithelial wall.^16^ Despite the increase in pro-inflammatory bacteria, we did not see an increase in IgA coated bacteria; surprisingly, there was a trend toward less IgA coating. Further, fecal lipocalin-2, as a marker for intestinal inflammation,^21^ did not change after surgery. Other features of the intestinal barrier (e.g., mucus layer) could reduce the coating of IgA and the contact of bacteria with the epithelium. For example, a dense and thick mucus layer on top of the epithelium might prevent bacteria from being coated or inducing an immune response. It has been noted in animal studies that obese mice have a less intact mucus layer.^37^ The mucus layer harbors major portions of IgA.^38^ When intact, bacteria are less able to interact with the epithelium, and IgA coating is less likely. Although not tested in humans, there is a higher mucus production in animals after bariatric surgery.^39,40^ This might also explain why some bacteria, such as *E. coli* and *S. salivarius,* are enriched in the IgA coated fraction before surgery, but not significantly enriched in either of the fraction after RYGB surgery.

*A. muciniphila* is an important driver of a healthy intestinal mucus layer by promoting mucus production and thickness.^41^ In the present study, we report an increase in *A. muciniphila* after surgery, which would support the notion of an improved mucus thickness after bariatric surgery. Interestingly, *A. muciniphila* is IgA coated in several studies.^24^ This bacterium is a potent mucus degrader, which in turn enhances the mucus production. However, by degrading mucus, *A. muciniphila* comes close to the intestine and might induce a strong IgA response. Furthermore, its LPS structure is highly pro-inflammatory.^28^ Therefore, this bacterium is beneficial for the host, but needs controlling to avoid inflammation. Other beneficial bacterial groups, such as *Bacteroides, Roseburia*, and *Faecalibacterium* are mostly uncoated. There might be numerous reasons: For example, the host might promote beneficial bacteria and those bacteria are not in close contact with the epithelial wall.^42,43^

We speculate that there are three drivers of IgA coating. First, proximity to the intestinal wall plays a major role in determining whether an inflammatory response is initiated (e.g. *A. muciniphila*). Second, bacteria in the small intestine are more coated with IgA^44^ (Figure S8), potentially due to a thinner mucus layer compared to the large intestine^45^ (e.g., *Streptococcus*^46^) and a major production of IgA in the small intestine.^47^ Third, colonic bacteria are mainly uncoated due to the thick mucus layer and their niche in the lumen (e.g., *Roseburia and Faecalibacterium*^46^).

We need to acknowledge that our study shows several limitations. First, we show the relative abundance of bacteria; if one bacterial strain is expanding, then another one will decrease, which is not an indication for its absolute abundance. To further explore the concept of IgA coating, it would be interesting to measure the total abundance of these bacteria in either of the fractions instead of relative abundances. Second, we used TLR reporter cells that might not be very specific to small amounts of exogenous (bacterial) ligands, particularly in complex samples, such as blood or fecal samples. Thirdly, we cultured B cells at two different time points that were one year apart. Differences between time points in *ex vivo* cultures might therefore been subjected to technical artifacts (e.g., lot numbers, biological activity of cytokines, etc.).

In conclusion, in the present study we show that the anatomical change of intestine after RYGB surgery is associated with a shift toward a more pro-inflammatory gut microbiome. After RYGB surgery, we, however, observed a lower systemic inflammatory tone, with subtle differences between non-diabetic controls and T2D individuals. This can be explained by an improved intestinal barrier function, characterized by a compensatory IgA response, particularly against pro-inflammatory compounds such as LPS. Thereby, it prevents further systemic inflammation driven by the gut microbiome.

## Methods

### Data availability

The data that support the findings of this study are available in ENA at https://www.ebi.ac.uk/ena/browser/view/PRJEB48095, reference number PRJEB48095.

### Study population

This work was carried out on a subsample from the BARIA cohort (Amsterdam UMC and Spaarne Gasthuis, The Netherlands).^9^ The study protocols were approved by the Ethical Review Board of the Academic Medical Center, Amsterdam (approval code: NL55755.018.15), and all patients that have been included provided informed consent. In the present study, we included 20 obese individuals with Type 2 diabetes mellitus and 20 body-mass-index (BMI), sex and age matched controls. Individuals underwent RYGB (laparoscopic omega‐loop gastric bypass or laparoscopic sleeve gastrectomy). Samples were collected before RYGB surgery as well as a year after surgery. A 2-hour mixed meal test was performed after an overnight fast with two Nutridrinks compact 125 mL (Nutricia®) as described.^9^

### Biochemical analysis

Blood was collected in Vacutainer® tubes containing a polymer gel for serum separation (Beckton Dickinson, Franklin Lakes, NJ), centrifuged at 1550 × g (15 min, 4°C) and stored at −80°C until further analysis.

### Antibody ELISAs

Total antibodies were measured in serum or fecal water according to the manufacturer’s instructions (Total IgG, IgA, and IgM Human Uncoated ELISA kit, ThermoFisher Scientific, US).

### LPS and Flagellin specific antibody ELISA

Antibodies were measured according to Tran, Ley, Gewirtz, and Chassaing.^48^ A 96-well half-area ELISA microplate (ThermoFisher Scientific, US) were coated with 50 uL flagellin (100 ng per well in 9.6 pH bicarbonate buffer; Invivogen, US) overnight at 4°C. Plates were washed 3x with 150 uL PBS with 0.05% Tween20 and were loaded with 50 uL of diluted serum samples (100x diluted in PBS) for 1 hours at 37°C. Plates were washed again and loaded with secondary antibodies for 1 h at room temperature (HRP-conjugated anti-human IgG, BD, clone G18-145, 2500x diluted in PBS with 0.05% Tween20; goat HRP-conjugated anti-human IgA, preadsorbed, abcam, polyclonal, 10.000× diluted; goat HRP-conjugated anti-human IgM, preadsorbed, polyclonal, abcam, 50.000× diluted). Plates were washed again and incubated with 50 uL TMB for 15 minutes and stopped with 50 uL 2 M H_2_SO_4_. Antibody activity was measured by reading OD450.

### Flagellin and LPS detection

Flagellin and LPS were detected in serum and fecal water with the aid of HEK-blue^TM^ TLR5 or HEK-blue^TM^ TLR4 reporter cell line (InvivoGen, US), according to the manufacturer's instructions. For serum flagellin and LPS detection, 20 uL serum was mixed with 180 uL cell suspension.

### Fecal IgA flow cytometry and sorting of IgA+ bacteria

Frozen fecal samples (100 mg) were diluted in 1:10 phosphate buffered saline (PBS) with cOmpleteTM Protease Inhibitory Cocktail (Roche, Basel, Switzerland). All steps were performed on ice. Samples were homogenized with the aid of a 5 mm stainless steel bead (Qiagen, US). Samples were centrifuged at 400 × g for 5 min to separate the bacteria from large debris. Next, 100 μL of the supernatant was taken and centrifuged for 5 min at 8000 × g to pellet the bacteria. Supernatant was saved for total antibody concentrations (fecal water). The pellet was washed with 1 mL of PBS and used for bacterial sorting as well as sequencing of total fecal bacteria.

Bacterial sorting was performed according to Palm et al (2014)^49^ with some modifications. Fecal bacteria were prepared as described above. Bacteria were washed with 1 mL PBS containing 1% Bovine Serum Albumin (BSA, Sigma-Aldrich, US, staining buffer). Pellets were blocked in a 100 uL blocking buffer (20% normal mouse serum in staining buffer, Jackson Immuno Research, UK) for 20 min on ice and then stained with 100 uL PE-conjugated anti-human IgA in staining buffer (1:50; Miltenyi Biotec, clone IS11-8E10, Germany) for 30 min on ice. Samples were washed 3 times with 1 mL staining buffer before cell separation. Anti-IgA stained fecal bacteria were incubated in a 1 mL staining buffer containing 50 uL anti-PE magnetic activated cell sorting (MACS) beads (Miltenyi Biotec, Germany) for 15 min at 4°C. Next, the bacteria were washed twice with a 1 mL staining buffer (10.000 × g, 5 min, 4°C) and separated with an LS column on a manual separator (Miltenyi Biotec, Germany). Both positive and negative fractions were further purified with SH800 cell sorter (Sony, Japan). For each sample, 2 million bacteria were collected, pelleted (10,000 × g, 5 min, 4°C) and frozen for DNA isolation. Gating strategies can be found in supplementary Figure S6.

### DNA Extraction and sequencing analysis of IgA-coated bacteria

DNA was extracted from 100 uL of homogenized fecal bacteria (see above) and the sorted fractions using a repeated bead beating protocol^50^ (method 5). DNA was purified using Maxwell RSC Whole Blood DNA Kit. 16S rRNA gene amplicons were generated using a single-step PCR protocol targeting the V3-V4 region.^51^ PCR products were purified using Ampure XP beads and purified products were equimolar pooled. The libraries were sequenced using a MiSeq platform using the V3 chemistry with 2 × 251 cycles.

Forward and reverse reads were truncated to 240 and 210 bases, respectively, and merged using USEARCH.^52^ Merged reads that did not pass the Illumina chastity filter, had an expected error rate higher than 2, or were shorter than 380 bases were filtered. Amplified Sequence Variants (ASVs) were inferred for each sample individually with a minimum abundance of 4 reads.^53^ Unfiltered reads were then mapped against the collective ASV set to determine the abundances. Taxonomy was assigned using the RDP classifier^54^ and SILVA^55^ 16S ribosomal database V132. Contaminants were identified using decontam software and subsequently, together with lab-specific known contaminants, removed from the data. Raw sequence reads were submitted to the ENA repository under study PRJEB47045.

### B cell isolation and culture

Peripheral blood mononuclear cells (PBMCs) were isolated from 10 mL fresh blood with Lymphoprep density gradient (Stemcell Technologies, Canada), according to manufacturer’s instructions. B cells were enriched for CD19+ cells with magnetic beads and MS columns on a manual system (Miltenyi Biotec, Germany). CD19+ cells were stained with conjugated antibodies against CD20-BV510 (BD, US), CD27-PE (BD, US), IgG-FITC (BD, US), IgA-APC (Miltenyi Biotec, German), and sorted on a Sony SH800 sorter (Japan) in full B cell media (RPMI 1640, 5% FBS, 1x Pen/Strep, 1x L-Glutamine, 50 µM β mercaptoethanol). The gating strategy is depicted in Figure S7.

Four hours before co-culture, 24 well plates were prepared with irradiated 3t3-CD40L feeder cells (30 Gy, 40.000 wells per well, full B cell media). CD19+ CD20+ were plated separately in a 24 well plate according to their antibody isotype (IgA+, IgG+, or IgM+, 4000 cells per well). Cells were cultured for 6 days and harvested by separated cells from the supernatant (300× g, 5 minutes). Cells were lysed with 300 µL RIPA buffer (ThermoFisher, US). Samples were frozen until further use.

### Statistical analysis

Data were checked for normality with the Shapiro-Wilk test. The effects of surgeryon fasting parameters were assessed using the paired t-test for normal continuous variables and the Wilcoxon signed rank test for other variables. A one-way ANOVA for repeated measures of normal continuous variables and the Friedman test for other variables, with Bonferroni post-hoc testing, were used to assess the effects of the high-fat meal on postprandial parameters.Statistical analysis was performed using GraphPad Prism version 8.0.2. Data are provided as mean with standard deviation. Microbiome data were analyzed and visualized in R^56^ (V3.6.3). Permutation ANOVA from Vegan^57^ was used to test differences in composition. Multilevel PCA was performed, on clr transformed data, using the mixOmics^58^ package, statistical significance was tested using a permutation manova on the first 10 components. The effects of sorting on specific taxa were tested with linear mixed effect models using lme4.^59^ P-values were corrected for multiple testing (FDR), were applicable, and P-values <0.05 were considered statistically significant. All authors had access to the study data and reviewed and approved the final manuscript.

## Supplementary Material

Supplemental Material
